# Comment on Oldrini et al. PHILOS Synthesis for Proximal Humerus Fractures Has High Complications and Reintervention Rates: A Systematic Review and Meta-Analysis. *Life* 2022, *12*, 311

**DOI:** 10.3390/life12081281

**Published:** 2022-08-22

**Authors:** Konstantinos Sidiropoulos, Konstantinos Tsikopoulos

**Affiliations:** 1 Orthopaedic Department, “Mamatsio” General Hospital of Kozani, 501 00 Kozani, Greece; 2 Orthopaedic Department, Queen Elizabeth University Hospital, Glasgow G51 4TF, UK

We read the paper published by Oldrini, L.M. et al. (2022) [1] with great interest. The paper is a systematic review and meta-analysis addressing a crucial topic regarding the complications and reinterventions in patients who have been surgically treated with PHILOS plates for proximal humerus fracture fixation. In this study, the authors concluded that the use of PHILOS plates leads to high complication and reintervention rates and suggested future authors compare the advantages and disadvantages in relation to the other solutions to manage patients with proximal humerus fractures. However, we would like to raise some issues that require clarification.

First of all, we wish to underline that there are no citations for the vast majority of the papers included in the meta-analysis. In particular, only 21 out of the 78 included studies have been cited, which represents not only a technical flaw but also represents a potential ethical concern. Regarding the inclusion and exclusion criteria, only studies with at least 12 months of follow-up were included. However, six studies have unknown or less than a year of follow-up (Aliudin et al., George et al., Moonot et al., Olerud et al., Trepat et al., Xue et al.). Moreover, as stated by the authors, one study did not report the number of complications, while the purpose of the meta-analysis was to “quantify and critically analyze the rate of complications”. Given the above, this paper should have been excluded from the systematic review.

We believe that all of the included studies used standard PHILOS plates, but we have been unable to identify a definition of “long PHILOS” in the text. In addition, the authors stated that data extraction was included in the study design characteristics, but there was no information about this in the paper. We note that distinguishing the level of evidence included in the source articles is of major clinical importance. To illustrate, drawing conclusions from evidence stemming from case series is completely different from interpreting results from randomized evidence. Accordingly, the quality appraisal using Down’s and Black’s tool yielded poor or fair classification scores for 57 out of 78 studies, which might have compromised the validity of the findings of this paper. In other words, the potentially significant bias in this study has not been commented on, as the reliability of the results is affected by the inclusion of poor-quality evidence more often than not.

Regarding the statistical analysis, we wish to point out some points that need further clarification. To begin with, information on the heterogeneity of the population was mentioned but was not adequately justified. As a result, the potential of the pooling data and the synthesis of the included studies remains unproven. On top of that, from a methodological point of view, a subgroup analysis to account for the different fracture types and the ages of the participants could have been appropriate. To elaborate further, an increase in complication rates is expected as age raises, and fracture types become more complex. As far as we are concerned, in the present study, the mean age of the participants mentioned in each and every original study was taken as a reference point. However, the distribution of this source of clinical heterogeneity remains unclear. We believe that the above unaddressed issues in addition to the complex structure of the Results section might confuse readers.

What is more, although conducting a subgroup analysis based upon the utilized surgical approach and fracture type provided useful information, other crucial parameters such as the age of the patients were not taken into consideration. There were 13 articles with a patient population over 55 years old included in the study, in which 347 complications were described. The confidence intervals were ample and were not mentioned for each of the presented complications.

Last but not least, in the Discussion section, no reference was provided to support the statement “Avascular Necrosis (AVN) of the humeral head is the most dangerous complication and one of the greatest concerns for the surgeon because it necessarily implies a reintervention”. Of note, the review published by Ayyash et al. (2021) [2] concludes that the early stages of AVN could be treated conservatively, while later stages (4 and 5) may require surgical interventions such as reverse shoulder arthroplasty or hemiarthroplasty. We would like to draw readers’ attention to the fact that the cause of AVN is not necessarily the surgical intervention, but is instead the fracture personality [3]. More specifically, displaced fractures are implicated in the majority of AVN cases, and their incidence can be as high as 21% and 75% for Neer type 3 and 4 fractures, respectively [4].
